# Response to Dethier et al, “Experience in nine additional cases of apremilast for therapy-resistant folliculitis decalvans”

**DOI:** 10.1016/j.jdcr.2024.12.001

**Published:** 2024-12-07

**Authors:** Pierre A. de Viragh, Seyed Morteza Seyed Jafari

**Affiliations:** Department of Dermatology, Inselspital, Bern University Hospital, Bern, Switzerland

**Keywords:** apremilast, folliculitis decalvans, therapy

*To the Editor:* We thank Dethier et al^1^ for sharing their experience of a difficult case of folliculitis decalvans in sustained remission after discontinuation of apremilast therapy. Over the last 4 years, we have extended our experience with apremilast in a further 9 cases of folliculitis decalvans refractory to conventional systemic and topical therapies. In 3 patients, we observed blunt treatment failure; in another case, a good initial response was reversed by relapsing while receiving treatment. In 3 patients, remission is maintained only with sustained therapy at conventional dose of 30 mg twice per day, accompanied by other anti-inflammatory therapies that are not sufficient on their own. In 1 patient, the dose can be quartered to 30 mg once daily every other day, reducing costs, but at 30 mg twice a week, folliculitis decalvans relapses. Finally, 1 patient shows sustained remission, as in the case of Dethier et al,^1^ requiring only minimal topical anti-inflammatory maintenance treatment. We suspect on clinical grounds that the inadequate effect of apremilast in some may be the result of pathogenic aspects of lichen planopilaris in the individual’s disease, also described as the “folliculitis decalvans and lichen planopilaris phenotypic spectrum.”^2^^,^^3^ Given the heterogeneity in folliculitis decalvans it is therefore not surprising that apremilast is not successful in all, but it is confirmed as a worthy addition to the armamentarium for difficult cases, in our hands and in the hands of others.

## Conflicts of interest

None disclosed.
